# Supplementary material for: Computational Study of Network and Type-I Functional Divergence in Alcohol Dehydrogenase Enzymes Across Species Using Molecular Dynamics Simulation
Source: Biomolecules. 2024 Nov 19;14(11):1473. doi: 10.3390/biom14111473 (PMC11591965; doi:10.3390/biom14111473)
Supplement: Supplementary file 1 [file biomolecules-14-01473-s001.zip › biomolecules-3263909-supplementary.pdf]

biomolecules

MDPI

Supplementary Materials

Computational Study of Network and Type-I Functional Divergence in Alcohol Dehydrogenase Enzymes Across Species Using Molecular Dynamics Simulation

Suhyun Park [1], Petrina Jebamani [2], Yeon Gyo Seo [1] and Sangwook Wu [1,3,*]

[1] Department of Physics, Pukyong National University, Busan 48513, Republic of Korea; suhyun@pukyong.ac.kr (S.P.); yeongs0709@pukyong.ac.kr (Y.G.S.)
[2] Department of Chemical Engineering, Pusan National University, Busan 46241, Republic of Korea; petrina@pusan.ac.kr
[3] PharmCADD, 1102-ho, 60, Centum Jungang-ro, Haeundae-gu, Busan 48059, Republic of Korea
* Correspondence: sangwoow@pknu.ac.kr; Tel.: +82-051-629-5549

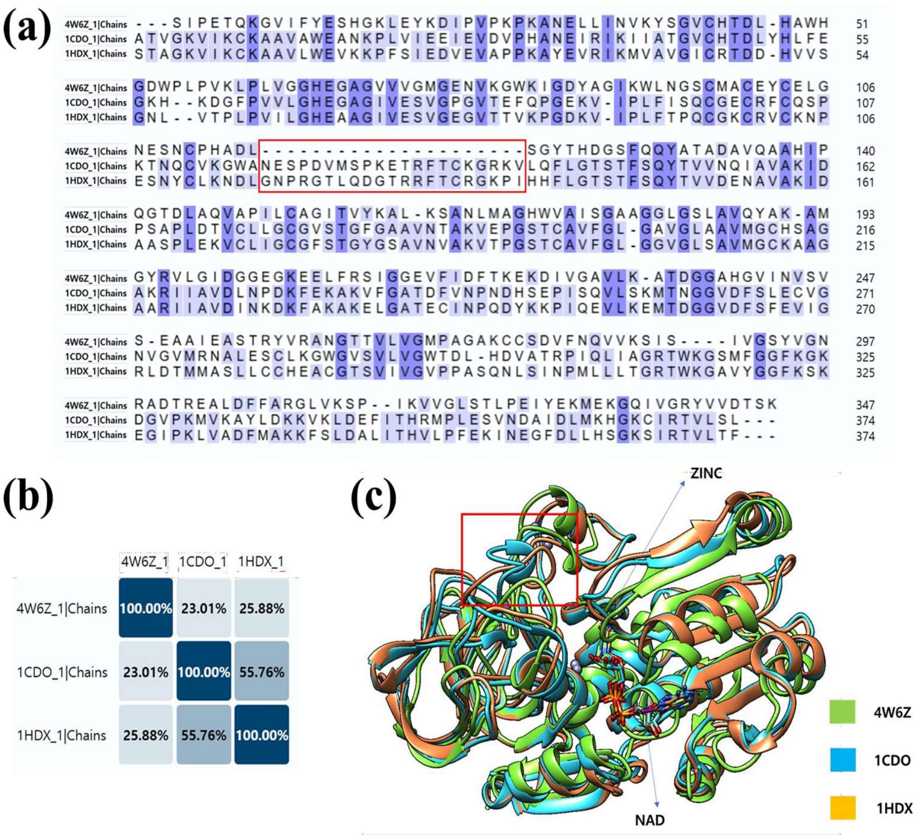

Figure S1. Sequence alignment and structure alignment for three PDB structures. (**a**) The sequence alignments for three PDB structures. (**b**) Identity matrix between sequences by UniProt Align module. The higher similarity is indicated by a darker color. (**c**) The sequences in the red box (**a**) are shown in the aligned structures. The zinc ions and NAD are structurally well conserved.

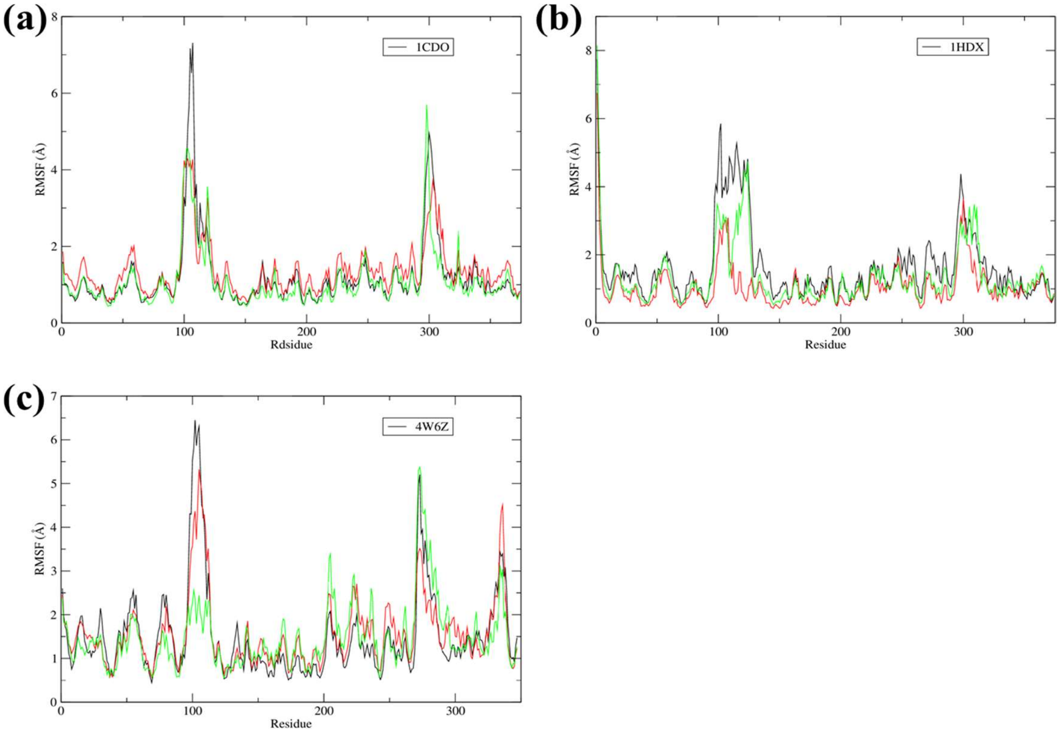

Figure S2. Root mean square fluctuation (RMSF) graphs during 100 ns MD simulations repeated three times for three PDB structures. (a) RMSF values during 100 ns MD simulations for 1CDO. (b) RMSF values during 100 ns MD simulations for 1HDX. (c) RMSF values during 100 ns MD simulations for 4W6Z.

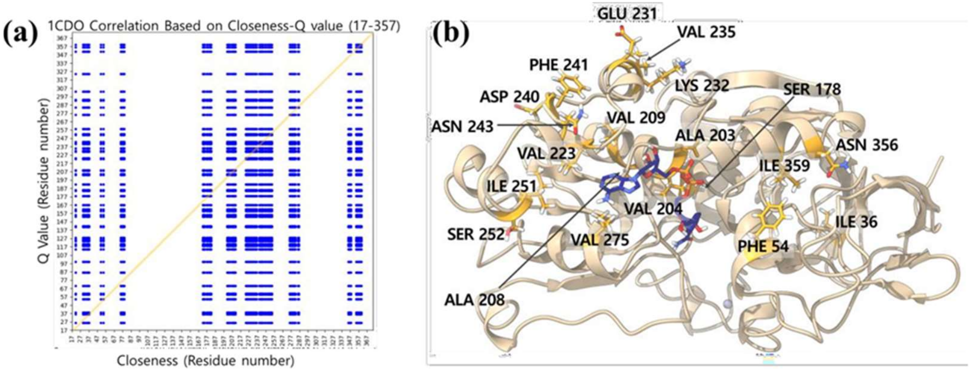

Figure S3. Correlation between Q-value and centrality measure for fish ADH1/ADH3 divergence (ADH1: 1CDO). (a) Q value plotted against closeness centrality values. (b) Key residues with matching closeness centrality and Q-value are highlighted and labelled.

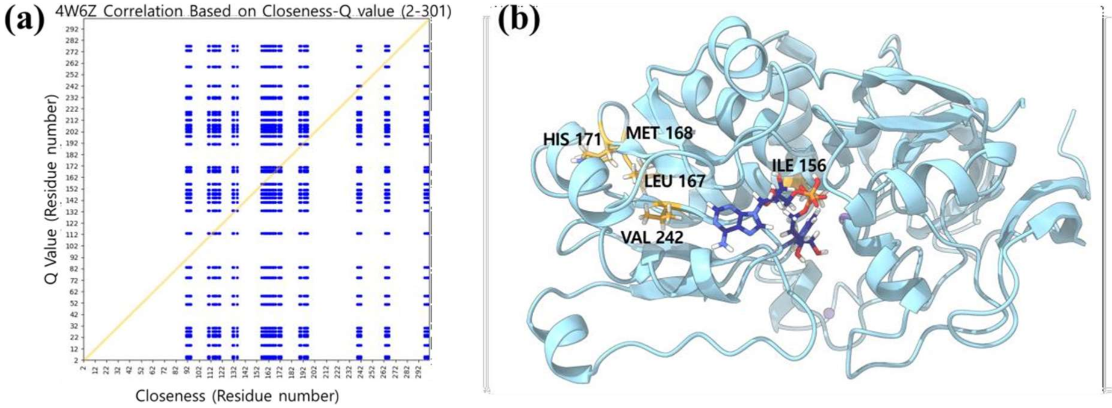

Figure S4. Correlation between Q-value and centrality measure for fungi ADH1/ADH5 divergence (ADH1: 4W6Z). (**a**) Q value plotted against closeness centrality values. (**b**) Key residues with matching closeness centrality and Q-value are highlighted and labelled.

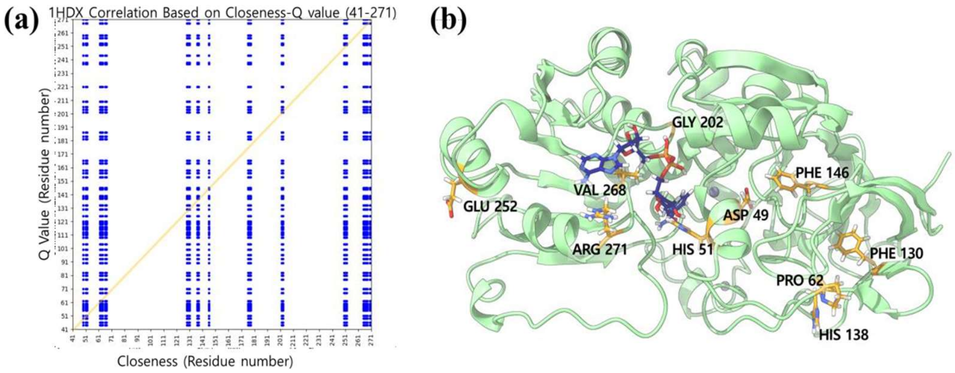

Figure S5. Correlation between Q-value and centrality measure for the Mammalia ADH1/ADH4 divergence (ADH1: 1HDX). (**a**) Q value plotted against closeness centrality values. (**b**) Key residues with matching closeness centrality and Q- value are highlighted and labelled.
